# Stainless Steel 304 and Carbon Mild Steel A36 Activity in Chloride-Containing Hybrid Pumice-Portland Cement Extract Pore Environment

**DOI:** 10.3390/ma18061216

**Published:** 2025-03-09

**Authors:** David Bonfil, Lucien Veleva, Jose Ivan Escalante-Garcia

**Affiliations:** 1 Applied Physics Department, Center for Research and Advanced Study (Cinvestav), Campus Merida, Merida 97310, Mexico; david.bonfil@cinvestav.mx; 2 Center for Research and Advanced Study (Cinvestav), Campus Saltillo, Ramos Arizpe 25900, Mexico; ivan.escalante@cinvestav.edu.mx

**Keywords:** hybrid pumice–Portland cement, cement extract solution, marine environment, chlorides, immersion test, corrosion potential, SEM-EDS, XPS, EIS

## Abstract

The effect of chlorides on the corrosion activities of SS304 and carbon steel A36 was investigated during immersion in a hybrid pumice–Portland cement extract solution, containing high concentration of chlorides (5 g $L^{-1}$ NaCl), in order to simulate the concrete–pore marine environment. The hybrid pumice–Portland cement (HB1) has been considered an alternative “green” cement system. The initial pH of the extract (12.99) decreased to 9.5 after 14 days, inducing a severe corrosion risk for A36, as suggested by the very negative corrosion potential (OCP ≈ −363 mV). Meanwhile, the SS304 tended to passivate and its OCP shifted to positive values (≈+72 mV). Consequently, the surface of the A36 presented a corrosion layer mainly of FeOOH, while that of the SS304 was composed of ${\mathrm{Cr}}_{2}O_{3}$, ${\mathrm{Fe}}_{3}O_{4}$ and NiO, according to the SEM-EDS and XPS analysis. An extended area of an almost uniform corrosion attack was observed on the A36 surface, due to the less protective Fe-corrosion products, while the SS304 surface presented some small pits of ≈1 µm. Based on electrochemical impedance measurements, the polarization resistance (Rp) and thickness of the passive layer were calculated. The Rp of the SS304 surface increased by two orders of magnitude up to ≈11,080 $k\Omega \;{\mathrm{cm}}^{2}$, and the thickness of the layer reached ≈1.5 nm after 30 days of immersion. The Rp of carbon steel was ≈2.5 $k\Omega \;{\mathrm{cm}}^{2}\;$ due to the less protective properties of its corrosion products.

## 1. Introduction

Traditional Portland cement (PC) concrete is characterized by its high alkaline pore solution (12.5<pH<14), which results from the formation of Ca(OH)_2_, KOH, and NaOH during the hydration process [1], promoting the formation of a thermodynamically stable layer of Fe-oxides/hydroxides on the carbon steel surface, which is known as the passive layer [2]. In stainless steel, this passive layer is characterized by the presence of a more protective and combined Fe/Cr-oxide layer [3,4]. The passive layers protect the reinforcing steel from corrosion in non-aggressive environments, provided that the pH of the concrete remains relatively stable. However, in aggressive industrial and marine environments, the reinforcing steel could be affected by concrete carbonation (loss of alkalinity) or/and ingress of chloride ions through concrete pores. Such a process can lead to the breakdown of the passive layer at the steel–concrete interface, known as the depassivation process, via localized attacks. The mechanism of stainless steel passivity breakdown due to ${\mathrm{Cl}}^{-}$ ions has been ascribed to several factors, proposed by several reported theories [5]. It has been suggested that ${\mathrm{Cl}}^{-}$ penetrates the passive film without destroying it (due to an electrostatic field), leading to the metal dissolution once the chloride ions reach the steel surface [6]. Another considered explanation is that pit nucleation occurs after the breakdown of the passive film, because of the electrostatic stress. One theory highlights the local thinning of the passive film, thus promoting steel pitting or localized corrosion. Some researchers argue that the adsorbed ${\mathrm{Cl}}^{-}$ ions form soluble intermediate complexes with the Fe-cations of the oxide film, causing the partial dissolution of the passive film [7]. If pitting nucleation succeeds as a metastable process, followed later by stable growth, at this stage, the breakdown is controlled by a diffusion process [8]. However, this process can be prevented by a repassivation event, mainly due to the formation of ${\mathrm{Cr}}_{2}O_{3}$ in the stainless steel passive film. On the carbon steel surface, depassivation results in the formation of thick Fe corrosion products that generate internal mechanical (tensile) stresses, causing rupture and detachment of the concrete cover and compromising the mechanical performance and durability of the reinforced concrete structures [9]. Coastal marine environments encompassing ≈356,000 km of coastline worldwide are particularly aggressive towards reinforced concrete structures, as classified by ISO 9223:2012 [10]. For instance, in Mexico, the coastal zone of the peninsula of Yucatan (Caribbean area) is categorized as highly corrosive, while that of the Veracruz port (Gulf of Mexico) is classified as very highly corrosive [11].

The steel–concrete interface may experience chloride-induced corrosion, when the ${\mathrm{Cl}}^{-}$ concentration reaches a threshold value ranging from 0.04% to 8.34 wt.% for PC systems [12,13,14]. This threshold depends on several factors, including the cement chemical composition, the chloride-binding capacity of the hydration products, the concrete density, the steel’s composition (including the presence of metallic inclusions in the steel matrix), and the availability and diffusion of $O_{2}$ through the concrete pores.

In recent years, the increase in PC clinker manufacturing has been associated with concerns related to the environment. The accelerated growth of global urbanization has led to more extensive consumption of PC clinker, reaching ≈4.4 Mt/year in 2021 [15], and estimated to increase to ≈5000 Mt/year by 2050 [16]. The manufacturing process of PC requires large quantities of natural resources and substantial amounts of energy (fossil fuels) to reach up to 1400 °C in the kiln. The chemical decomposition of limestone during this process emits significant amounts of CO_2_, contributing to 8–10% of global ${\mathrm{CO}}_{2}$ emissions [17,18]. To counteract this environmental problem, alternative “green” and sustainable cements have been developed and characterized, some examples of which are alkali-activated, blended, supersulfated, and hybrid cements, among others [19]. These alternative cements involve the partial or total replacement of the PC with supplementary cementitious materials (SCMs), including natural pozzolan (volcanic materials), limestone, metakaolin, blast furnace slag, fly ash, calcinated clays and waste glass, whose composition is within the ternary diagram SiO_2_-CaO-Al_2_O_3_ [20,21,22,23,24]. Some studies have reported that alternative cement systems are suitable for marine environments because of the refinement of the pore structure, which favors a denser concrete microstructure that reduces the diffusion of ${\mathrm{Cl}}^{-}$ ions and air oxygen [25].

However, by reducing or substituting the PC with SCMs, the cement hydration mechanism can be altered, leading to variations in the chemical composition of the concrete pore solution. These variations include changes in alkalinity (pH), ionic strength, conductivity (resistivity), which are associated with different concentrations of Na, Al, Si, and S ionic species, depending on the composition of the precursors and activators used [26]. The reduction in the pH alkalinity is associated with a reduction in the alkali content in the cement and the pozzolanic reactions that consume a part of the alkali reserve for the formation i.e., C-S-H and C-A-S-H cement hydration products [27,28]. Alkali-activated cements exhibit higher carbonation rates due to their lower pH buffer capacity attributed to the lower $\mathrm{CaO}/{\mathrm{SiO}}_{2}$ ratio in the hydration products (C-S-H) [29,30].

To prevent some experimental difficulties in corrosion studies of steels embedded in concrete, model solutions of saturated Ca(OH)_2_ (pH ≈ 12.6), NaOH, and KOH solutions (or mixtures of them) have been proposed as more representative examples with which to investigate the electrochemical response of steels, simulating the concrete pore at the metal surface [31,32,33,34,35,36,37]. Cement extract solutions offer an alternative testing approach, providing a closer approximation of the chemical composition of concrete pore environments as a function of the variety of ions present [38,39,40]. Current studies on the effect of pore solution composition on steel passivation focus on PC systems and alkali-activated cements. However, there is a lack of knowledge regarding steel corrosion behavior in other alternative “green” cement pore environments.

The relation between free chlorides and bound chlorides is termed the chloride-binding capacity [41]. For example, when SCMs are used, the decrease in tricalcium aluminate C3A and the alkalinity of the concrete leads to a decrease in its chloride-binding capacity. This in turn results in an increase in free chlorides in the pore solution, which are detrimental to the corrosion resistance of the passive layer formed on the reinforcing steel surface [42,43,44]. Conversely, studies have reported that SCMs with high ${\mathrm{Al}}_{2}O_{3}$ content improve the chloride-binding capacity [45,46,47,48]. Some reports have indicated that the extracted pore solution of a blended cement with microsilica (10–20 wt.%) shows a pH reduction from 13.5 to 12.5 and an increase in the free chloride ion concentration [49]. Consequently, very high ${\mathrm{Cl}}^{-}$/${\mathrm{OH}}^{-}$ ratios increase the risk of corrosion of steel reinforcements. A simulated concrete pore solution of an alkali-activated slag (containing sulfur, aluminate, and silicate species) with the addition of NaCl was used to research the corrosion of hot-rolled ribbed steel bars of HRB400 [50]. The results revealed that the steel surface tended to passivate, which was attributed to the formation of a protective film of aluminates and silicates. In another report, the ionic composition of the leached pore solution of a calcium sulfoaluminate cement (pH ≈ 12.7) consisted mainly of ${\mathrm{Na}}^{+}$, $K^{+}$, ${{\mathrm{SO}}_{4}}^{2-}$ and small amount of ${\mathrm{Ca}}^{2+}$ content, while the ${\mathrm{OH}}^{-}$ (55.34 mmol L^−1^) was lower compared to the PC pore solution (451.3 mmol L^−1^) [51]. The higher contents of released ${{\mathrm{Al}(\mathrm{OH})}_{4}}^{-}$ ions may offer sufficient buffer capacity for the solution’s alkalinity. However, based on free corrosion potential measurements (OCP ≈ −675 mV), the passive film stability of the hot ribbed steel HRB400 surface was negatively affected by the presence of ${{\mathrm{SO}}_{4}}^{2-}$ for concentrations of ${\mathrm{OH}}^{-}$ ≤ 0.01 M. In the presence of chlorides, the high ${{\mathrm{SO}}_{4}}^{2-}$ content in the simulated calcium sulfoaluminate cement pore solution deteriorated the passive film of the HRB400 carbon steel, elevating its sensitivity to the localized chloride pitting attack. It was suggested that the ${\mathrm{OH}}^{-}$ released from the hydrolysis of ${{\mathrm{Al}(\mathrm{OH})}_{4}}^{-}$ may inhibit the local acidification in the chloride-induced corrosion pit. In the meantime, the precipitation of insoluble species within the corrosion pits acted as a physical barrier, hindering mass transport and the diffusion of oxygen and aggressive species [52]. On the other hand, the presence of ${{\mathrm{SO}}_{4}}^{2-}$ induced the loss of corrosion products’ formation, leaving the steel unprotected from ${\mathrm{Cl}}^{-}$ pitting attack [53]. Thus, the effect of different ions present in alternative cement’s pore solutions can influence steel passivation, depending on the ${\mathrm{OH}}^{-}$ concentration (pH value). At higher pH values, the adsorption of ${\mathrm{Cl}}^{-}$ and ${{\mathrm{SO}}_{4}}^{2-}$ ions on the passive film is reduced, and the corrosion resistance increases; in contrast, at low pH values, even low concentrations of ${\mathrm{Cl}}^{-}$ and ${{\mathrm{SO}}_{4}}^{2-}$ can induce corrosion in low-carbon steels. At high pH values in the presence of ${\mathrm{Cl}}^{-}$ and ${{\mathrm{SO}}_{4}}^{2-}$, the corrosion risk is mitigated due to the competitive absorption of both ions onto the steel interface [54].

In order to simulate concrete–pore environments, previous studies have characterized “green” cement water extracts, including supersulfated pumice–Portland cement (SSC, pH = 12.3), sodium silicate-modified limestone–Portland cement (JLSC1, pH = 12.6), and hybrid pumice–Portland cement (HB1), have been characterized. The water extracts of these cements showed an increase in their ${{\mathrm{SO}}_{4}}^{2-}$ ion concentration, which was attributed to the activators, along with greater concentrations of Na, K, and Si, as well as ${\mathrm{Cl}}^{-}$ ions from the pumice for SSC and HB1 [55,56,57,58]. The electrochemical behavior and surface change of carbon steels A36 and B450C, as well as low-chromium ferritic stainless steel SS430 and SS304, were examined after exposure to the cement extract solutions. The preliminary results were compared with those observed during exposure to the Portland cement extract. The initial pH of the HB1 cement extract was 12.99, attributed mainly to the formation of NaOH during the curing process, which induced the passivation of A36 and SS304 [58]. However, as the pH dropped, the A36 showed an intermediate risk of corrosion, according to the free corrosion potential (OCP) values. Meanwhile, electrochemical impedance (EIS) data suggested that the passive layer formed in HB1 cement extract was thicker, providing greater corrosion resistance to the steel than that formed in the PC cement extract.

HB1 cement is a formulation with a lower PC content, substituted by volcanic pumice (45.82%) and activated with ${\mathrm{Na}}_{2}{\mathrm{SO}}_{4}$ and ${\mathrm{Ca}(\mathrm{OH})}_{2}$. Given its good passivation properties [58], this study investigates the effect of the chloride ions on the corrosion performance of carbon steel A36 and stainless steel SS304 during exposure to hybrid pumice–Portland cement extract containing 5 g $L^{-1}$ NaCl, labelled as “HB1 + Cl”, to simulate a coastal marine environment. The changes in pH and free corrosion potential (OCP) were monitored over 30 days. Electrochemical impedance spectroscopy (EIS) Nyquist and Bode diagrams were registered to characterize the steel interface/HB1 + Cl extract solution. The steel surfaces were characterized by X-ray photoelectron spectroscopy (XPS) to identify the composition of the corrosion layers formed. SEM-EDS analyses were carried out to verify the corrosion attack on the steel surfaces.

## 2. Materials and Methods

### 2.1. Steel Samples

Flat rectangular samples of austenitic stainless steel SS304 (Outokumpu Mexinox, San Luis Potosi, SLP, Mexico) and mild carbon steel A36 (Steeland, Jalisco, GDL, Mexico), with nominal compositions reported in Table 1, were cut to dimensions of 20 × 20 × 1 mm. The surfaces were wet-grounded using SiC abrasive paper with progressively finer grit sizes (600, 800, 1000, 2000, and 4000) and absolute ethanol, sonicated for 10 min in ethanol (Branson 1510, Branson Ultrasonics Co., Danbury, CT, USA), and dried at 21 °C. The steel samples were stored in sealed containers with paraffin tape and left in a desiccator until the immersion test.

### 2.2. Hybrid Cement HB1 and HB1 + 5 g L^−1^ NaCl

The hybrid cement system, labeled as HB1, consists of 45.82% volcanic pumice, 45.82% PC (CPC30R) as precursors, and 5.82% ${\mathrm{Na}}_{2}{\mathrm{SO}}_{4}$ and 2.87% calcium hydroxide ${\mathrm{Ca}(\mathrm{OH})}_{2}$ as alkaline activators, in a molar ratio of ${\mathrm{Na}}_{2}{\mathrm{SO}}_{4}/{\mathrm{Ca}(\mathrm{OH})}_{2}=1$ [57]. The main variations in the oxide composition of HB1 cement compared to PC cement (Table 2) are a reduction in CaO contents in HB1 by approximately half; a doubling of those of ${\mathrm{SiO}}_{2}$, ${\mathrm{Al}}_{2}O_{3}$, and ${\mathrm{SO}}_{3}$; and concentrations of ${\mathrm{Na}}_{2}O$, $K_{2}O$ ≈ 15 that are increased by eight times. These changes are ascribed to the activators and the pumice’s composition [57].

The cement extract of HB1 was prepared with ultrapure deionized water (18.2 MΩ cm) in a cement/water ratio of 1. The stirred mixture was left to hydrate for 24 h (hydration) in a closed recipient to avoid carbonation. Then, 5 g $L^{-1}$ (reagent grade) was added to the filtered supernatant (2.5 µm pore size filter paper, Whatman, Kent, UK), and the resulting extract solution, labelled as “HB1 + Cl”, was stored in a sealed container. The chemical ionic composition of the HB1 + Cl cement extract, determined using absorption spectrometry and atomic emission by plasma analysis, is presented in Table 3. The free ${\mathrm{Cl}}^{-}$ ion concentration was determined using an ion-selective electrode. The HB1 + Cl cement extract was predominantly composed of ${\mathrm{Na}}^{+}$, $K^{+}$, ${\mathrm{Ca}}^{2+}$, ${{\mathrm{SO}}_{4}}^{2-}$ and ${\mathrm{OH}}^{-}$ due to its sulphatic and alkaline environment, which induced the dissolution of the volcanic pumice and the precipitation of hydration products [58]. During the hydration of PC calcium silicates, the formed ${\mathrm{Ca}(\mathrm{OH})}_{2}$ reacted with the ${\mathrm{Na}}_{2}{\mathrm{SO}}_{4}$ activator via cationic exchange (Equation (1)) [59], leading to the formation of NaOH, which increased the alkalinity of the HB1 + Cl cement extract. Furthermore, the higher $K_{2}O$ and ${\mathrm{Na}}_{2}O$ contents (Table 2) in the volcanic pumice also contributed to an increase in the alkalinity of the HB1 + Cl extract solution.

(1) $${\mathrm{Na}}_{2}{\mathrm{SO}}_{4}+{\mathrm{Ca}(\mathrm{OH})}_{2}\to {\mathrm{CaSO}}_{4}+\mathrm{NaOH}$$

### 2.3. Immersion Tests

The immersion tests were conducted on the steel samples according to the ASTM-NACE/ASTM G31-12a standard [60]. Triplicated samples of A36 and SS304 steels (0.8 ${\mathrm{cm}}^{2}$ of the working area) were immersed in 10 mL of HB1 + Cl cement extract for 30 days in sealed containers using paraffin tape. The pH of the HB1 + Cl was measured after 1, 7, 14, 21, and 30 days of immersion (PH60 Premium Line, pH tester, Apera Instruments, LLC., Columbus, OH, USA). After a 30-day period, the samples were withdrawn and dried at room temperature. The surface morphology and composition of the corrosion products were analyzed with SEM-EDS (XL-30 ESEM-JEOL JSM-7600F, JEOL, Ltd., Tokyo, Japan) and XPS (K-Alpha, Thermo Scientific, Waltham, MA, USA) at different times of erosion with a scanning Ar-ion gun. Subsequentlty, the corrosion product layers were removed, according to the ASTM G1-03 standard [61], and the underlying steel surfaces were analyzed by SEM-EDS to asses the corrosion attack.

### 2.4. Electrochemical Measurements

Electrochemical measurements were performed using a conventional three-electrode cell configuration inside a Faraday cage. The setup consisted of a saturated calomel electrode (SCE, CH Instruments Inc., Austin, TX, USA) as the reference, a platinum mesh as the auxiliary, and the A36 and SS304 steel samples as working electrodes (0.8 ${\mathrm{cm}}^{2}$). The electrodes were connected to a potentiostat/galvanostat (Interface-/ZRA, 1000EGamry instruments, Warminster, PA, USA). The open-circuit potential (OCP), also known as the free metal corrosion potential, was registered after 1, 7, 14, 21, and 30 days of immersion of the steel samples in HB1 + Cl cement extract solution. Nyquist and Bode diagrams of the electrochemical impedance spectroscopy (EIS) were produced in the frequency range from 100 kHz to 10 mHz, with an AC signal of ±10 mV at the OCP; a total of 10 data points per decade were recorded for samples after 1, 7, 14, 21, and 30 days of immersion. V. 7.1 Gamry Echem Analyst software (Philadelphia, PA, USA) was used to analyze the EIS data.

## 3. Results

### 3.1. Change in pH of HB1 + Cl Cement Extract and Steel Corrosion Potential 

Table 4 presents the change in pH of HB1 + Cl cement extract solution and the free corrosion potential (OCP, mV vs. SHE) of the A36 carbon steel and SS304 stainless steel samples after 30 days of immersion testing. To reveal the effect of the chloride ions on steel activity, the values were compared (Table 4) with those obtained in HB1 cement extract solution [58].

The initial pH of the compared cement extracts was 12.99, and it decreased gradually, reaching an almost stable value at 30 days ≈ 9.60 in HB1 + Cl and ≈9.07 in HB1 (Table 4). It was reported that at pH > 10, the direct reaction between ${\mathrm{OH}}^{-}$ and a small fraction of aqueous carbon dioxide predominated the hydration, causing a decrease in pH and contributing to the release of ${{\mathrm{HCO}}_{3}}^{-}$ ions [62]. When the pH dropped below 10, the rate of the direct hydration mechanism decreased, and the carbonate/bicarbonate buffer almost stabilized the pH of the solution due to ${\mathrm{OH}}^{-}$ being continuously regenerated by the hydrolysis of ${{\mathrm{CO}}_{3}}^{2-}$ [63,64].

The change in pH values influenced the activity of the steels in different ways, the indication of which is the change in time of their free corrosion potential values (Table 4). In the HB1 cement extract, the initial negative OCP value of the carbon steel A36 (≈−89 mV) moved positively at 30 days (≈88 mV), indicating that it reached a passive state (Table 4). Meanwhile, the A36 could not be passivated in the presence of ${\mathrm{Cl}}^{-}$ (HB1 + Cl), and the OCP shifted to very negative values (≈−363 mV), meaning it was exposed to a severe corrosion risk (ASTM C876) and active corrosion of the A36 surface. The effect of ${\mathrm{Cl}}^{-}$ ions, among other anions such as ${{\mathrm{SO}}_{4}}^{2-}$, at different pH values has been reported for a variety of carbon steels and the dissolution of iron [65,66,67,68,69,70,71]. On the other hand, the austenitic SS304 could reach a passive state in the presence of ${\mathrm{Cl}}^{-}$ ions (HB1 + Cl), the OCP value of which (Table 4) was positive (≈72 mV), although less than that obtained in HB1 (≈265 mV).

### 3.2. Stainless Steel 304 Surface Characterization After Immersion in HB1 + Cl Cement Extract 

**Figure 1:**
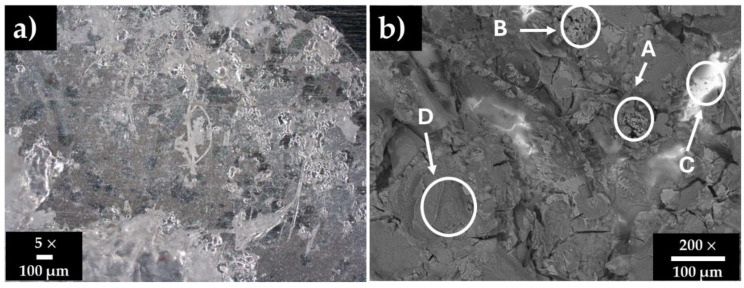
Stainless steel 304 surface images after 30 days of exposure to HB1 + Cl cement extract solution: (**a**) optical microscope image and (**b**) SEM micrograph.

Figure 1 shows surface images of the stainless steel SS304 after 30 days of immersion in HB1 + Cl cement extract, and Table 5 presents the corresponding EDS analysis. After 30 days (OCP ≈ +72 mV and pH ≈ 9.6, Table 4), SS304 kept its passive state, and its surface was almost covered with a layer of deposits. The EDS analysis (Figure 1b) in zones A, B, and D presented high contents of O, S, Ca, Na, K, and Cl, indicating the precipitation of sulfates, carbonates, and NaCl (zone B). ${\mathrm{Cl}}^{-}$ anions may accumulate over time at the passive layer–solution interface, inducing the degradation of the passive film by localized (pitting) attacks (zone B); however, the pits will not be able to grow any further, due to the competitive adsorption of ${{\mathrm{SO}}_{4}}^{2-}$ ions (acting as pitting inhibitors) [72]. In the meantime, zone C corresponded to the matrix of the steel, not yet covered by deposits.

**Figure 2:**
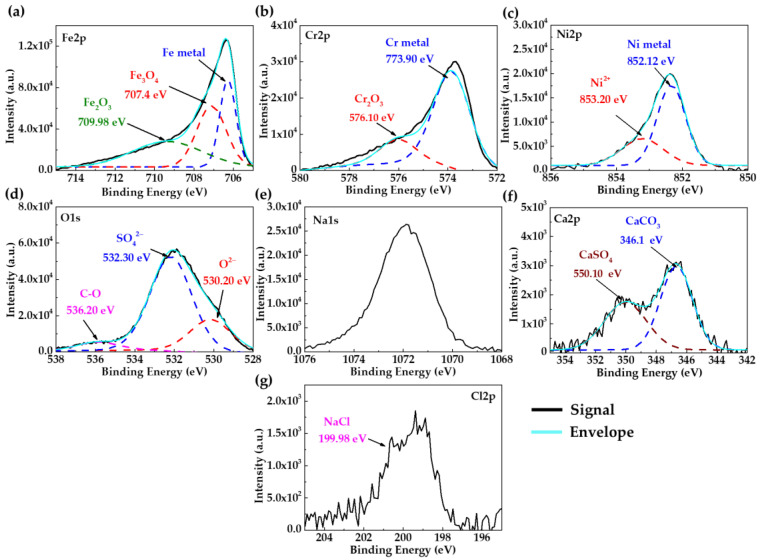
XPS spectrum of SS304 surface after 30 days of immersion in HB1 + Cl cement extract: (**a**) Fe2p; (**b**) Cr2p; (**c**) Ni2p; (**d**) O1s; (**e**) Na1s; (**f**) Ca2p; (**g**) Cl2p. This spectrum corresponds to 580 s of erosion with a scanning Ar-ion gun.

As additional surface information, Figure 2 presents the XPS spectra of the SS304 after 30 days of immersion in HB1 + Cl cement extract solution. The deconvolution of Fe2p (Figure 2a), Cr2p (Figure 2b), and Ni2p (Figure 2c) indicates that the passive layer is composed of ${\mathrm{Fe}}_{3}O_{4}$ (707.40 eV), ${\mathrm{Fe}}_{2}O_{3}$ (709.98 eV); ${\mathrm{Cr}}_{2}O_{3}$ (776.10 eV) [73,74,75], and NiO (853.20 eV) [76]. The deconvolution of O1s (Figure 2d) shows three characteristic peaks attributed to $O^{2-}$ (530.2 eV), ${{\mathrm{SO}}_{4}}^{2-}$ (532.30 eV), and ${{\mathrm{CO}}_{3}}^{2-}$ (535.80 eV) [77,78]; these peaks were associated with Na1s (Figure 2e) and Ca2p (Figure 2f), confirming the precipitation of sulphates and carbonates suggested by the SEM-EDS analysis. In addition, the presence of Cl2p (Figure 2g) was ascribed to NaCl (199.98 eV). The passive layer of stainless steel has been reported as a complex multilayer structure; the inner layer is enriched with ${\mathrm{Cr}}_{2}O_{3}$ and ${\mathrm{Fe}}^{2+}$ oxides, while the outer is rich in ${\mathrm{Fe}}^{3+}$ species (${\mathrm{Fe}}_{2}O_{3})$ due to the oxidation of ${\mathrm{Fe}}_{3}O_{4}$, a process facilitated by the pH decrease [79]. The Ni enrichment at the metal/oxide interface has been explained by the selective oxidation of iron and chromium compared to that of nickel or by the dissolution of nickel oxides [80]. It has been considered that the presence of Ni promotes the formation of ${\mathrm{Fe}}_{3}O_{4}$, which plays an important role in reducing susceptibility to pitting [81,82].

### 3.3. Carbon Steel A36 Surface Characterization After Immersion in HB1 + Cl Cement Extract 

**Figure 3:**
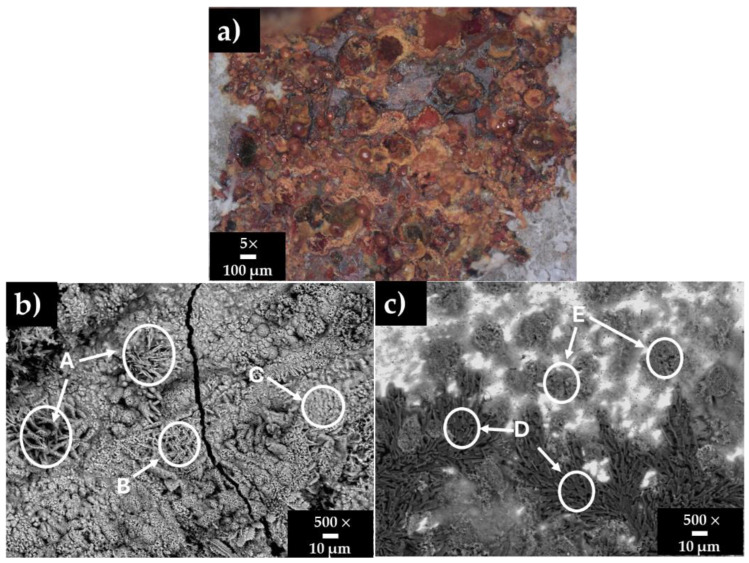
Carbon steel A36 surface images after 30 days of exposure to HB1 + Cl cement extract: (**a**) optical microscopy; (**b**) SEM micrograph of the corrosion products in the orange-brown (**a**) zone; (**c**) SEM micrograph of the deposits in the white (**a**) zone.

Figure 3 shows surface images of the carbon steel A36 after 30 days of immersion in HB1 + Cl cement extract, and Table 6 presents the corresponding EDS analysis. The OCP ≈ −363 mV and pH ≈ 9 indicated that A36 was at a severe risk of corrosion. Two distinct zones can be observed (Figure 3a): a corroded cracked area (orange-brown zone) and areas presenting the accumulation of deposits (white zones). The EDS analysis of the corroded zones A, B, and C (Figure 3b) revealed high contents of Fe and O, associated with variation in the morphology of different phases of the Fe corrosion products (Fe oxides/oxyhydroxides) in the presence of chlorides (zones B and C). It is reported that the corrosion layer formed on the carbon steel surface is composed of an inner ${\mathrm{Fe}}_{3}O_{4}$ oxide layer, which further tends to be oxidized to ${\mathrm{Fe}}^{3+}$ species, such as ${\mathrm{Fe}}_{2}O_{3}$ (hematite), and different phases of oxyhydroxides, α-FeOOH (goethite), β-FeOOH (akageneite), and γ-FeOOH (lepidocrocite) [83]. According to the literature, several morphological structures can be distinguished in the oxide layers: small crystalline globules or fine plates “flowery or nesty structures” (γ-lepidocrocite); globular structures known as cotton balls (goethite); and dark flat regions with circular disks (magnetite) [84,85,86]. α-FeOOH and γ-FeOOH have been considered the most common phases of the corrosion of carbon steel, while the formation of the β-FeOOH has been reported for environments with a high concentration of chlorides [87,88]. On the other hand, the EDS analysis of zones of deposits (E-D, Figure 3c) suggests that they are compounded mainly by Na-sulphates and carbonates.

**Figure 4:**
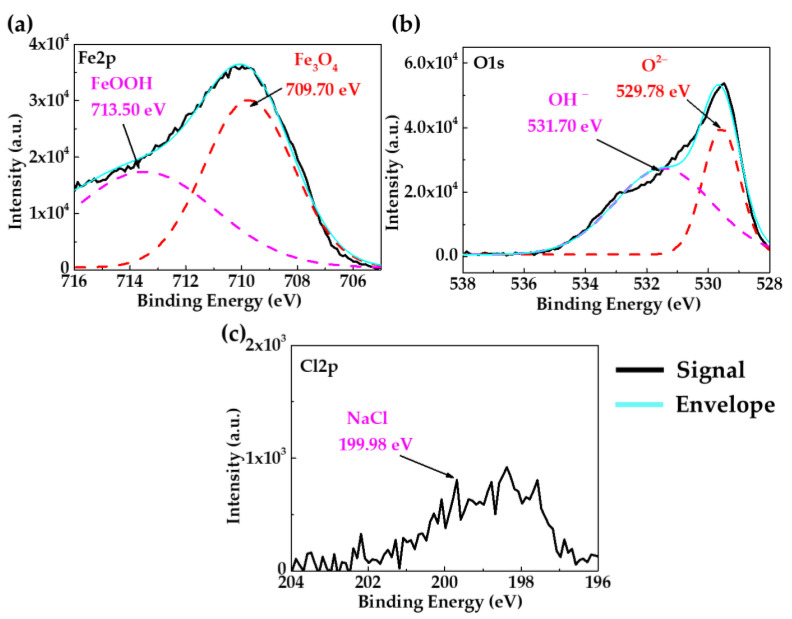
XPS spectrum of the surface of carbon steel A36 (orange-brown zone) after 30 days of immersion in HB1 + Cl cement extract: (**a**) Fe2p; (**b**) O1s; (**c**) Cl2p. (This spectrum corresponds to 580 s of erosion with a scanning Ar-ion gun).

**Figure 5:**
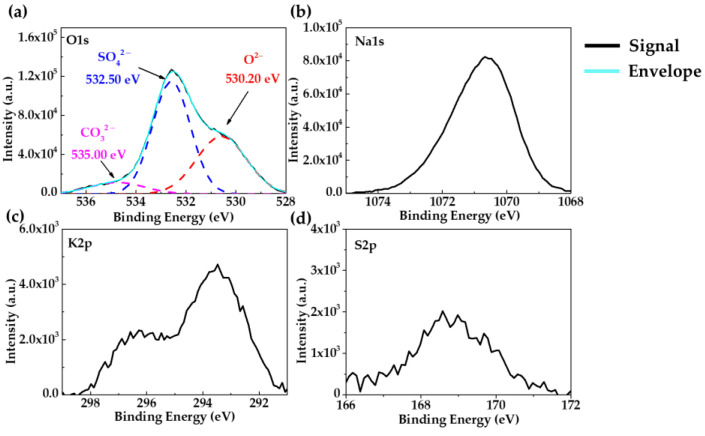
XPS spectrum of the surface of carbon steel A36 (white zone) after 30 days of immersion in HB1 + Cl cement extract: (**a**) O1s; (**b**) Na1s; (**c**) K2p; (**d**) S2p. (This spectrum corresponds to 580 s of erosion with a scanning Ar-ion gun).

XPS spectra (Figure 4) provided additional information on the layer composition of the orange-brown zone (Figure 3a) on the A36 carbon steel surface. The deconvoluted peaks of Fe2p (Figure 4a) and O1s (Figure 4b) indicated that the corrosion layer is composed of ${\mathrm{Fe}}_{3}O_{4}$ (709.7 eV), which is a combination of ${\mathrm{Fe}}^{2+}$ and ${\mathrm{Fe}}^{3+}$ iron states and FeOOH (713.50 eV) [89,90]. The peak of Cl2p at 199.98 eV (Figure 4c) was detected in this zone. The presence of chlorides in the marine environment may affect the atomic structure of the corrosion products, inducing the transformation of ${\mathrm{Fe}}^{2+}$ oxides to ${\mathrm{Fe}}^{3+}$ (${\mathrm{Fe}}_{2}O_{3}$ and FeOOH), which is considered less protective [90,91]. A mechanism for the formation of Fe-corrosion products has been proposed, suggesting that initially, the Fe oxidizes to ${\mathrm{Fe}}^{2+}$ to form FeO, later oxidizing to ${\mathrm{Fe}}^{3+}$, corresponding to the formation of $\mathrm{FeO}\cdot {\mathrm{Fe}}_{2}O_{3}$ (${\mathrm{Fe}}_{3}O_{4}$) and FeOOH [92]. In the meantime, the chloride ions act as stimulators of the iron corrosion process, leading to secondary reactions forming some intermediate products, such as FeCl, with a short lifetime. Moreover, they are less dense and voluminous, allowing the diffusion of aggressive species such as $O_{2}$ and ${\mathrm{Cl}}^{-}$ to the steel surface. According to the XPS spectra (Figure 5), the deconvoluted peak of O1s (Figure 5a) indicated the presence of sulphates ${{\mathrm{SO}}_{4}}^{2-}$ (at 532.50 eV) and carbonates ${{\mathrm{CO}}_{3}}^{2-}$ (at 535.00 eV) [77,78], the composition of which may be ascribed to the white-colored deposits observed in Figure 3c (zones E-D). The peaks of Na1s (Figure 5b), K2p (Figure 5c), and S2p (Figure 5d) were attributed to the different crystals (Table 6).

### 3.4. Steel Surface Damage After Immersion Tests in HB1 + Cl Cement Extract 

**Figure 6:**
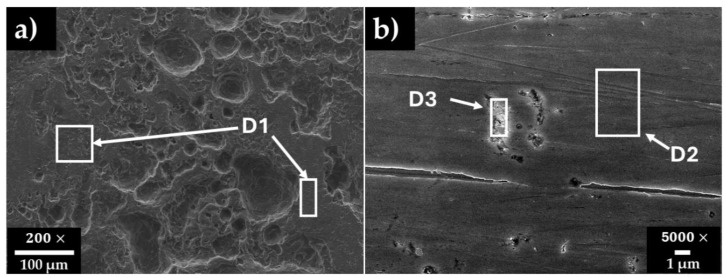
SEM micrographs of steel surfaces after the removal of the layer formed during the exposure over 30 days to HB1 + Cl cement extract solution: (**a**) carbon steel A36 (×200) and (**b**) stainless steel SS304 (×2000).

Figure 6 shows the SEM micrographs of (a) carbon steel A36 and (b) stainless steel SS304 surfaces after the chemical removal of the layers formed during the exposure to the HB1 + Cl cement extract for 30 days. Table 7 presents the EDS respective surface analysis.

On the carbon steel A36 surface, an extended area of corrosion attacks was observed (Figure 6a), suggesting an almost uniform corrosion, although in separate zones, the presence of pits of ≈10–100 µm was observed. These facts are in agreement with the shift in the corrosion potential to negative values (Table 4) and the pH to lower alkalinity, as an indication of severe risk of corrosion. The formed non-stable and less protective corrosion layer of FeOOH and ${\mathrm{Fe}}_{3}O_{4}$ on the carbon steel surface allowed the continuous penetration of aggressive ions such ${\mathrm{Cl}}^{-},$ which were present in the cement extract solution, as well as the diffusion of the $O_{2}$ in the air. The EDS analysis (Table 7, Figure 6a, zone D1), indicated that the steel matrix, composed mainly of Fe and a low content of C, may be ascribed to the presence of local Fe carbides, acting as active cathodes (reducing the $O_{2}$); thus, the pits may occur in their surroundings (localized corrosion). The pits observed on the carbon steel exposed to the HB1 extract solution [58] were up to 25 times smaller in size than those observed in the HB1 + Cl extract solution (Figure 6a).

On the stainless steel SS304 surface (Figure 6b) only small pits of ≈1 µm were observed similar in size to those previously reported for HB1 [58]. Their formation occurred in the vicinity of Fe carbides and Mn-inclusions (Table 7, Figure 6b, zone D3), acting as active cathodes. It has been reported that due to the small size of ${\mathrm{Cl}}^{-}$ ions, these could penetrate the passive layer formed of ${\mathrm{Cr}}_{2}O_{3}$ and ${\mathrm{Fe}}_{3}O_{4}$ and contribute to the development of pits [93]. The high contents of Fe, Cr, and Ni (Table 7, Figure 6b) were associated with the Fe-Cr-Ni structure of the 304-grade steel. The presence of defects in the steel, such as inclusions, secondary phases, and dislocations, are recognized as initiation sites for pitting nucleation due to the presence of chloride ions [94,95].

### 3.5. Activity at the Steel–Cement Extract Interface: Nyquist and Bode EIS Diagrams 

**Figure 7:**
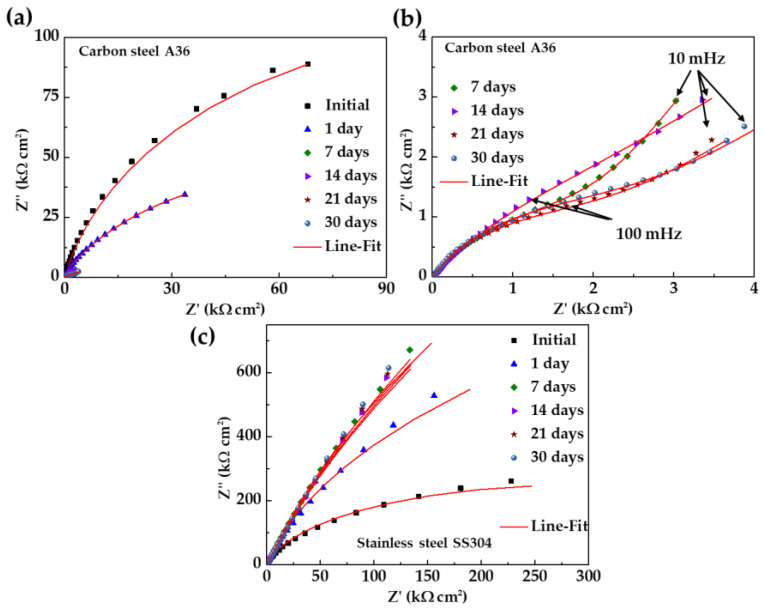
Nyquist impedance diagram of (**a**) carbon steel A36; (**b**) zoom of (**a**); and (**c**) SS304 with their fitting lines, during 30 days of immersion in HB1 + Cl cement extract solution.

Looking for the parameters that characterize the activity of the carbon and steel surfaces in contact with the extract cement solution HB1 + Cl (electrolyte), Nyquist diagrams of the electrochemical impedance (EIS) were produced (from 100 kHz to 10 mHz frequency). From the first day of immersion, the corrosion potential of the carbon steel A36 shifted to a more negative value (ending at −364 mV, Table 4), and this fact agrees with the incomplete depressed semi-circles presented by the Nyquist diagrams (Figure 7a,b). These circles tended to decrease, indicating that the steel surface was not able to develop a passive state because of the presence of ${\mathrm{Cl}}^{-}$ ions (2866 mg $L^{-1}$) (Table 3). Later, after 7 days of immersion, when the alkalinity of the solution decreased (pH ≈ 10, Table 4), a linear diffusion tail appeared in the low-frequency domain (10–100 mHz, Figure 7b) because of the non-stable and less protective corrosion layer that formed, allowing the continuous penetration of aggressive species (${\mathrm{Cl}}^{-}$ and $O_{2}$).

Meanwhile, at the initial time, the Nyquist diagram of the SS304 showed an incomplete depressed semi-circle (10–100 mHz), the Z″ values of which increased over time, changing to semi-linear diffusion impedance (Figure 7c). This fact confirmed the capacity of the stainless steel to develop a passive state, as was suggested by the shift in corrosion potential to positive/noble values (+72 mV, Table 4). It was reported that the Cr species are more stable than the Fe species at low alkaline values (pH ≈ 9), and the enrichment in oxides (Figure 2) densifies the passive film structure and reduces the conducting of charges due to the filling of cation vacancies by Cr [96,97].

**Figure 8:**
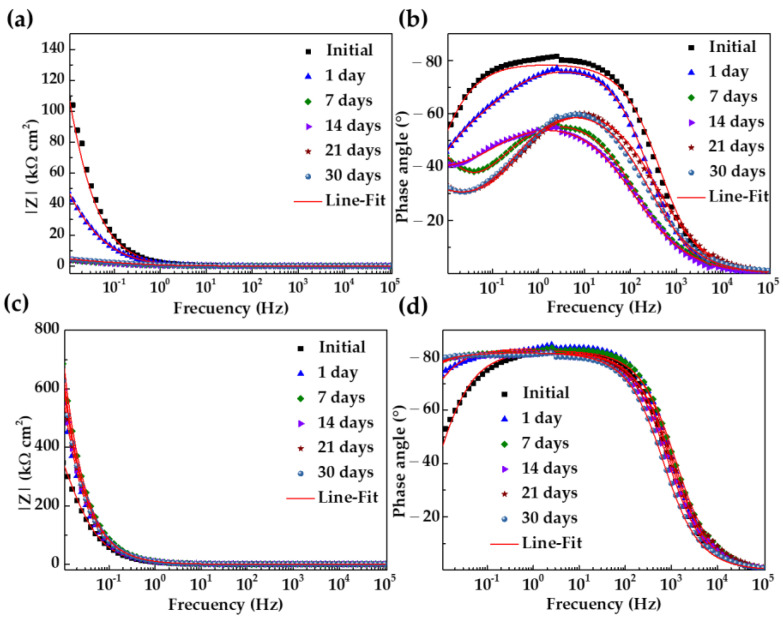
Bode impedance diagrams of (**a**,**b**) carbon steel A36 and (**c**,**d**) stainless steel SS304 with their fitting lines over 30 days of immersion in HB1 + Cl cement extract solution.

Figure 8 compares the Bode impedance diagrams of the carbon steel A36 (a,b) and SS304 (c,d) during their immersion in HB1 + Cl cement extract solution. The impedance module $\left|Z\right|$ for A36 (Figure 8a) tended to decrease, as an indication of the more active steel corrosion activity, which is influenced mainly by the presence of ${\mathrm{Cl}}^{-}$ and a shift of the atmosphere to a less alkaline pH (at 7–30 days). The initial value of the phase angle θ (Figure 8b) was ≈55° and decreased over time, which can be ascribed to the less protective properties of the corrosion layer formed on the carbon steel surface. Meanwhile, the impedance module $\left|Z\right|$ of the stainless steel increased to twice its initial value after 30 days of exposure (Figure 8c), and the corresponding phase angle θ (Figure 8d) also increased up to θ ≈ 80°, revealing that the passive layer acquired capacitive behavior due mainly to the presence of Cr oxides. Thus, on the stainless steel, an effective barrier to the ingress of aggressive species (${\mathrm{Cl}}^{-}$ and $O_{2}$) from the cement extract solution to the steel surface was grown.

**Figure 9:**
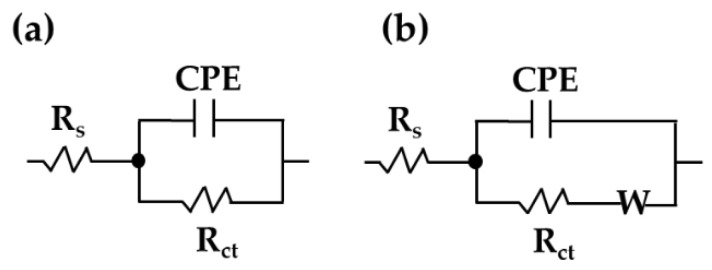
Equivalent circuit used for stainless steel 304 (**a**) and carbon steel A36 (**b**) to quantify the EIS data during the exposure of the steels to HB1 + Cl cement extract solution.

Two equivalent circuits were used to quantify the EIS data: a simplified Randles equivalent circuit for the stainless steel (Figure 9a) and a Randles equivalent circuit with a semi-infinite Warburg “W” diffusion element for the carbon steel (Figure 9b). The elements $R_{s}\;$ and $R_{\mathrm{ct}}$ represent the HB1 + Cl cement extract solution’s resistance, which depends on its pH and ionic composition in contact with the steel surface and the charge transfer resistance, respectively. Constant phase elements (CPEs) were used, considering the imperfect capacitive nature of the double-layer capacitance in the presence of a passive layer on the SS304 surface or the corrosion layer on the carbon steel A36 surface, both of which depend on their composition and porosity (and additionally on the surface substrate’s roughness). The Warburg element was introduced to characterize the specifics of the mass transport process occurring at the carbon steel–electrolyte interface in the low-frequency domain (Nyquist diagram, Figure 7b) due to the minimally protective properties of the carbon steel corrosion products, which allowed the diffusion of chemical species. The CPE values and their change in time reflect the exponential factor *n*, the range of which was 0–1. When *n* tends to 1, the CPE element behaves as a capacitor; when *n* ≈ 0.5, the process is controlled by diffusion; and when *n* tends to 0, the element exhibits resistive behavior [98].

Table 8 compares the fitting parameters obtained from the EIS diagrams; their goodness of fit, $\chi^{2},$ was good in most cases ($10^{-4}$). For SS304, the *n* parameter kept a constant value of ≈0.90 during the 30 days of immersion in the HB1 + Cl cement extract, corroborating the capacitive nature of the formed passive layer. Consequently, the polarization resistance value ($R_{p}$), which is an indicator of the stability of the passive layer, showed an increase up to 10,850 $k\Omega \;{\mathrm{cm}}^{2}$ after 14 days of exposure, in accordance with the pronounced increase in the corrosion potential up to positive values (Table 4, OCP) during this period. At 30 days of immersion, the $R_{p}$ decreased to ≈11,080 $k\Omega \;{\mathrm{cm}}^{2}$, suggesting the influence of the lower alkalinity (pH) of the cement extract solution and the lesser stability of the passive layer in the presence of ${\mathrm{Cl}}^{-}$.

For the carbon steel A36, the *n* decreased to a value of ≈0.66–0.70 due to the formation of the Fe-corrosion layers (Figure 3), having a very minimally capacitive nature. In the meantime, after 7 days of immersion, the $R_{p}$ presented low values of ≈2.5 $k\Omega \;{\mathrm{cm}}^{2}$, based on favorable charge transfer and transport of mass between the carbon steel matrix and the aggressive environment of HB1 + Cl.

To calculate the thickness (*d*) of the passive layers (Equation (2)) formed on the steel surfaces [99,100], the CPE values (Table 8) were transformed into effective capacitance (C), according to Equation (3) [101], considering that the passive layer is a homogeneous parallel plate capacitor:

(2) $$d=\frac{\varepsilon \varepsilon_{0}A}{C}$$

(3) $$C=\;{\mathrm{CPE}\;}^{\frac{1}{n}}{\left(\frac{R_{s}R_{\mathrm{ct}}}{R_{s}+{\;R}_{\mathrm{ct}}}\right)}^{\frac{1-n}{n}}$$

where $\varepsilon_{0}$ is the vacuum permittivity ($8.85\times 10^{-14}\;F\;{\mathrm{cm}}^{-1}$), A is the working area of the steel sample (${\mathrm{cm}}^{2}$), and ε is the dielectric constant, assumed to be 12 for carbon steel and 15.6 for stainless steel [102,103].

**Figure 10:**
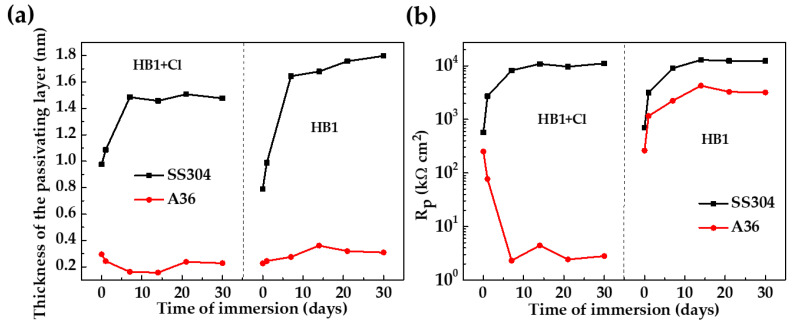
Comparison of (**a**) *d* of the passive layers formed and the (**b**) $R_{p}$ during the immersion in HB1 + Cl and HB1 cement extracts for 30 days.

Figure 10 compares the calculated *d* (Figure 10a) and $R_{p}$ (Figure 10b) of carbon steel A36 and SS304 during the immersion in HB1 + Cl cement extract with those in the HB1 cement extract [58]. After 7 days of immersion in HB1 + Cl, the stainless steel passive layer reached *d* ≈ 1.5 nm, keeping almost constant until 30 days (Figure 10a). This fact was associated mainly with the self-regeneration of the ${\mathrm{Cr}}_{2}O_{3}$ layer, even in the presence of chloride attacks, while the ${\mathrm{Fe}}_{3}O_{4}$ layer lost stability after more immersion time due to the decrease in the alkalinity (pH) of the cement extract solution. On the other hand, the $R_{p}$ of SS304 also tended to increase during its immersion in HB1 + Cl. However, the values of d and $R_{p}$ obtained in the HB1 + Cl cement extract were slightly lower compared to those during the exposure to HB1 (Figure 10).

For the carbon steel A36 immersed in HB1 + Cl, the *d* was ≈0.2 nm (Figure 10a) and the $R_{p}$ decreased to very low values (Figure 10b) when the corrosion started; the corrosion layer of FeOOH and ${\mathrm{Fe}}_{3}O_{4}$ had no passivation properties. However, in the HB1 cement extract, the $R_{p}$ was almost four orders of magnitude larger (Figure 10b) due to the passive layer’s properties (the thickness of which was ≈0.35 nm).

## 4. Conclusions

This study reports the effect of chloride ions on the corrosion activities of SS304 and carbon steel A36 exposed for 30 days to a hybrid pumice–Portland cement extract containing 5 g $L^{-1}$ NaCl (HB1 + Cl), in order to simulate the concrete–pore marine environment. The hybrid pumice–Portland cement (HB1) has been considered as an alternative cement system.

1. The ionic composition of the HB1 + Cl cement extract solution presented elevated contents of ${\mathrm{Na}}^{+}$, $K^{+}$, ${{\mathrm{SO}}_{4}}^{2-}{\mathrm{Na}}^{+},$ and ${\mathrm{Cl}}^{-}$ (2866 mg L^−1^), and its pH ≈ 12.99 was attributed to the formation of NaOH and KOH. The pH tended to decrease due to the ${\mathrm{CO}}_{2}$ air dissolution and stabilized, reaching ≈ 9.5 after 14 days due to the carbonate/bicarbonate buffer capacity.
2. Meanwhile, the corrosion potential (OCP) value of the SS304 increased to more positive/noble values (≈+72 mV), tending to form a passive film on the surface. However, the OCP of A36 carbon steel fell to very negative/active values (≈−363 mV), indicating a severe corrosion risk.
3. In accordance with the surface analysis (SEM-EDS, and XPS), the passive state of the SS304 was attributed to the formation of a layer of ${\mathrm{Cr}}_{2}O_{3}$, ${\mathrm{Fe}}_{3}O_{4}$, and NiO, while the carbon steel A36 presented FeOOH phases as its main corrosion products.
4. An extended area of a uniform corrosion attacks was observed on the A36’s surface, due to the less protective Fe-corrosion layer allowing the ingress of ${\mathrm{Cl}}^{-}$ and the diffusion of $O_{2}$ towards the steel matrix. Meanwhile, the SS304’s surface presented only small pits of ≈1 µm.
5. The quantitative parameters, based on the EIS diagrams, indicated that the SS304 polarization resistance (R_p_) increased by two orders of magnitude up to ≈11,080 $k\Omega \;{\mathrm{cm}}^{2},$ and the thickness of the passive layer was ≈1.5 nm after 30 days of immersion; meanwhile, these parameters for the A36 were ≈2.5 $k\Omega \;{\mathrm{cm}}^{2}$ and ≈0.2 nm, respectively, corroborating the active corrosion state of A36.
6. The effect of the high concentration of chlorides (2866 mg $L^{-1}$) on the corrosion activities of the studied steels could be considered a consequence of the competitive adsorption process between ${\mathrm{Cl}}^{-}$ and the high concentration of ${{\mathrm{SO}}_{4}}^{2-}$ in the HB1 cement system.
7. The reported results suggest that chloride-containing hybrid pumice–Portland cement extract, which simulates the marine environment, is highly corrosive toward carbon steel A36, while the passivating properties of SS304 remain unaffected.

## Figures and Tables

**Table 1:** Nominal composition (wt.%) of SS304 austenitic stainless steel and A36 mild carbon steel, according to manufacturers.

Element (wt.%)	C	Cr	P	S	Mn	Si	Ni	Fe
SS304	0.08	18.00	0.045	0.03	2.00	1.00	8.00	Balance
A36	0.0650	0.02	0.004	0.0020	0.41	0.02	-	Balance

Balance used to complete the total 100%.

**Table 2:** Oxide compositions (wt.%) of hybrid HB1 and Portland cement (PC).

Cement	SiO_2_	Al_2_O_3_	Fe_2_O_3_	CaO	MgO	SO_3_	K_2_O	Na_2_O	Cl	LOI
HB1	43.05	8.85	2.79	29.78	0.75	4.87	2.88	4.35	0.05	2.63
PC	22.30	4.62	2.44	58.42	1.92	2.20	0.35	0.28	-	3.62

Note: Volcanic pumice contains (wt.%) $K_{2}O$ (4.24), ${\mathrm{Na}}_{2}O$ (3.9), CaO (3.78) [57].

**Table 3:** Ionic composition (mg $L^{-1}$) of hybrid HB1 + Cl and PC extract solutions.

Element $(\mathrm{mg}\;L^{-1}$)	Li	$K^{+}$	${\mathrm{Na}}^{+}$	${\mathrm{Al}}^{3+}$	${\mathrm{Ca}}^{2+}$	Si	${{\mathrm{SO}}_{4}}^{2-}$	Sr	${\mathrm{Cl}}^{-}$	${\mathrm{OH}}^{-}$
HB1	1.32	2480.20	17,358.90	0.37	210.00	4.39	10,115.00	9.74	2866.20	1661.00
PC [47]	-	1373.58	420.71	-	256.51	-	-	-	-	3434.80

Note: ${\mathrm{OH}}^{-}$ was calculated from the pH; the content of chlorides includes 172 mg $L^{-1}$ as a part of HB1 (from the volcanic pumice).

**Table 4:** Change with time in the HB1 and HB1 + Cl cement extract solutions’ pH and the average values of free corrosion potential (OCP vs. SHE) of the tested steel samples after 30 days of immersion testing.

		Time (Days)	Initial	1	7	14	21	30
**HB1 + Cl**	SS304	pH	**12.99**	12.90	**10.37**	9.61	9.61	**9.60**
		OCP vs. SHE (mV)	**−191.484**	−87.98	**+8.29**	+49.31	+62.65	**+72.41**
	A36	pH	**12.99**	12.92	**10.50**	9.73	9.62	**9.61**
		OCP vs. SHE (mV)	**−144.85**	−288.29	**−359.12**	−386.88	−371.93	**−363.64**
**HB1**	SS304	pH	**12.99**	12.92	**10.48**	9.61	9.18	**9.07**
		OCP vs. SHE (mV)	**−204.89**	−89.81	**+128.126**	+219.75	+245.30	**+265.08**
	A36	pH	**12.99**	12.93	**10.11**	9.57	9.10	**9.08**
		OCP vs. SHE (mV)	**−89.05**	−24.84	**+48.58**	+67.456	+79.154	**+88.18**

**Table 5:** Stainless steel 304 EDS average analysis (wt.%) of the areas marked on the SEM images.

Zones/wt.%	Fe	Cr	Ni	O	Na	Ca	S	K	Si	C	Cl	Sr
A	-	-	-	**43.17**	2.88	**17.59**	0.93	1.83	2.87	**16.64**	2.31	13.77
B	-	-	-	**37.24**	**17.20**	**9.29**	**3.98**	**5.83**	**7.40**	-	**19.06**	-
C	**63.16**	**16.34**	**9.45**	4.21	-	-	-	-	2.70	2.75	-	-
D	-	-	-	51.57	27.44	-	12.46	0.66	3.85	2.58	0.98	-

**Table 6:** EDS average analysis (wt.%) of carbon steel A36 and the areas marked on the SEM images (Figure 3).

Zones/wt.%	Fe	O	Na	K	S	C	Si	Al	Cl
A	**47.18**	**46.34**	-	-	**1.79**	3.16	-	-	1.53
B	**43.00**	**49.03**	0.90	**-**	**0.88**	**3.77**	**-**	**-**	2.41
C	**48.06**	**45.53**	**1.91**	-	1.11	2.43	-		0.96
D	1.52	**59.31**	**25.93**	-	-	**10.78**	2.06	0.41	-
E	6.25	**44.86**	**23.28**	3.23	**11.66**	**4.06**	5.62	0.72	0.28

**Table 7:** EDS surface average analysis (wt.%) of carbon steel A36 and stainless steel SS304 of the market zones in the SEM images (Figure 6).

Steel/wt.%		Fe	Cr	Ni	Mn	Si	C
**A36**	D1	96.91	-	-	-	-	3.09
**SS304**	D2	65.34	18.17	15.29	1.21	-	-
	D3	54.57	16.27	11.55	5.96	6.68	6.67

**Table 8:** Fitting EIS parameters obtained for the SS304 and carbon steel A36 exposed to HB1 + Cl cement extract solution up to 30 days.

Steel	Days	$R_{s}$ $k\Omega \;{\mathrm{cm}}^{2}$	$R_{\mathrm{ct}}$ $k\Omega \;{\mathrm{cm}}^{2}$	CPE ${\mu \mathrm{Ss}}^{n}{\mathrm{cm}}^{-2}$	*n*	$R_{p}$ $k\Omega \;{\mathrm{cm}}^{2}$	$\chi^{2}$ $10^{-4}$
SS304	0	0.01	570.1	25.66	0.91	570.1	6.52
	1	0.01	2706.0	21.24	0.92	2706.0	7.88
	7	0.01	8203.0	17.51	0.91	8203.0	5.64
	14	0.02	10,850	19.95	0.90	10,850	1.95
	21	0.01	9651	19.00	0.90	9651	13.29
	30	0.02	11,080	18.56	0.90	11,080	9.32
A36	0	0.01	251.4	83.09	0.87	251.4	8.33
	1	0.01	77.04	124.7	0.84	77.04	1.10
	7	0.01	2.32	506.0	0.70	2.32	4.89
	14	0.01	4.43	699.4	0.66	4.43	1.81
	21	0.01	2.43	329.4	0.70	2.43	4.01
	30	0.01	2.81	314.7	0.70	2.81	3.93

Note: The polarization resistance values ($R_{p}$) are almost equal to the charge transfer resistance values, $R_{\mathrm{ct}}$ (minus $R_{s}$).

## Data Availability

The original contributions presented in this study are included in the article. Further inquiries can be directed to the corresponding author.

## References

[1] Neville A.M. (2009). Properties of Concrete.

[2] Pourbaix M. (1990). Thermodynamics and corrosion. Corros. Sci..

[3] Freire L., Carmezim M.J., Ferreira M.G.S., Montemor M.F. (2010). The passive behaviour of AISI 316 in alkaline media and the effect of pH: A combined electrochemical and analytical study. Electrochim. Acta.

[4] Yuan X., Wang X., Cao Y., Yang H. (2020). Natural passivation behavior and its influence on chloride-induced corrosion resistance of stainless steel in simulated concrete pore solution. J. Mater. Res. Technol..

[5] Szklarska-Smialowska Z. (2002). Mechanism of pit nucleation by electrical breakdown of the passive film. Corros. Sci..

[6] Evans U.R. (1927). The passivity of metals. Part I. The isolation of the protective film. J. Chem. Soc..

[7] Strehblow H.H., Marcus P., Oudar J. (1995). Corrosion Mechanism in Theory and Practice.

[8] Burstein G.T., Pistorius P.C., Mattin S.P. (1993). The nucleation and growth of corrosion pits on stainless steel. Corros. Sci..

[9] Rodriguez R., Gaboreau S., Gance J., Ignatiadis I., Betelu S. (2021). Reinforced concrete structures: A review of corrosion mechanisms and advances in electrical methods for corrosion monitoring. Constr. Build. Mater.

[10] (2012). Corrosion of metals and alloys–Corrosivity of atmospheres–Classification, Determination and Estimation.

[11] Millano-González V., Traconis de Rincón O., Torres Acosta A.A., Sanchez Gómez M.A., Castro-Borges P., Perez-Quiroz J.T., Pérez-López T., Vera R., Salta M., Pedrón M. (2023). Modeling Electrochemical Performance of Reinforced Concrete in Natural Marine Airborne-Exposure Environments: DURACON Project, 10-Year Evaluation. Corrosion.

[12] Glass G.K., Buenfeld N.R. (1997). The presentation of the chloride threshold level for corrosion of steel in concrete. Corros. Sci..

[13] Glass G.K., Reddy B., Buenfeld N.R. (2000). The participation of bound chloride in passive film breakdown on steel in concrete. Corros. Sci..

[14] Gouda V.K. (1970). Corrosion and corrosion inhibition of reinforcing steel: I. immersed in alkaline solutions. Br. Corros. J..

[15] Tanash A.O., Muthusamy K., Mat Yahaya F., Ismail M.A. (2023). Potential of recycled powder from clay Brick, sanitary ware, and concrete waste as a cement substitute for concrete: An overview. Constr. Build. Mater..

[16] Supriya, Chaudhury R., Sharma U., Thapliyal P.C., Singh L.P. (2023). Low-CO_2_ emission strategies to achieve net zero target in cement sector. J. Clean. Prod..

[17] Andrew R.M. (2018). Global CO_2_ Emissions from Cement Production. Earth Syst. Sci..

[18] Aitcin P.-C., Aïtcin P., Flatt R.J. (2016). Portland cement. Science and Technology of Concrete Admixtures.

[19] Juenger M.C.G., Winnefeld F., Provis J.L., Ideker J.H. (2011). Advances in alternative cementitious binders. Cem. Concr. Res..

[20] Ramezanianpour A.A. (2014). Cement Replacement Materials: Properties, Durability, Sustainability.

[21] Lothenbach B., Scrivener K., Hooton R.D. (2011). Supplementary cementitious material. Cem. Concr. Res..

[22] Juenger M.C.G., Snellings R., Bernal S. (2019). Supplementary cementitious materials: New sources, characterization and performance insights. Cem. Concr. Res..

[23] Játiva A., Etxeberria M. (2024). Exploring the Utilization of Activated Volcanic Ash as a Substitute for Portland Cement in Mortar Formulation: A Thorough Experimental Investigation. Materials.

[24] Wang Y., Shui Z., Gao X., Yu R., Huang Y., Cheng S. (2019). Understanding the chloride binding and diffusion behaviors of marine concrete based on Portland limestone cement-alumina enriched pozzolans. Constr. Build. Mater..

[25] Al-Amoudi Q.S.B. (2002). Durability of plain and blended cements in marine environments. Adv. Cem. Res..

[26] Juenger M.C.G., Siddique R. (2015). Recent advances in understanding the role of supplementary cementitious materials in concrete. Cem. Concr. Res..

[27] Canham I., Page C.L., Nixon P.J. (1987). Aspects of the pore solution chemistry of blended cements related to the control of alkali silica reaction. Cem. Concr. Res..

[28] Vollpratcht A., Lothenbach B., Snellings R., Haufe J. (2016). The pore solution of blended cements: A review. Mater. Struct..

[29] Aperador W., Mejía de Gutiérrez R., Bastidas D.M. (2009). Steel corrosion behaviour in carbonated alkali-activated slag concrete. Corros. Sci..

[30] Puertas F., Palacios M., Vázquez T. (2006). Carbonation process of alkali-activated slag mortars. J. Mater. Sci..

[31] Poursaee A. (2010). Corrosion of steel bars in saturated Ca(OH)_2_ and concrete pore solution. Concr. Res. Lett..

[32] Jiang H., Jin Z., Zhang X., Qian L., Zhou Z. (2023). The Effect of Temperatures on the Passivation Behavior of Q235 Steel in the Simulated Concrete Pore Solution. Materials.

[33] Li L., Sagüés A.A. (2001). Chloride corrosion threshold of reinforcing steel in alkaline solutions-open circuit immersion tests. Corrosion.

[34] Zhang F., Jinshan P., Changjian L. (2009). Localized corrosion behaviour of reinforcement steel in simulated concrete pore solution. Corros. Sci..

[35] Behera P.K., Misra S., Mondal K. (2020). Corrosion Behavior of Strained Rebar in Simulated Concrete Pore Solution. J. Mater. Eng. Perform..

[36] Pokorný P., Vacek V., Prodanovic N., Zabloudil A., Fojt J., Johánekm V. (2022). The Influence of Graded Amount of Potassium Permanganate on Corrosion of Hot-Dip Galvanized Steel in Simulated Concrete Pore Solutions. Materials.

[37] Zakroczymski T., Fan C.J., Szklarska-Smialowska Z. (1985). Kinetics of passive film formation on iron in 0.05M NaOH. J. Electrochem. Soc..

[38] Montemor M.F., Simoes A.M., Ferreira M.G. (1998). Analytical characterization of the passive film formed on steel in solutions simulating the concrete interstitial electrolyte. Corrosion.

[39] Veleva L., Alpuche-Aviles M.A., Graves-Brook M.K., Wipf D.O. (2002). Comparative cyclic voltammetry and surface analysis of passive films grown on stainless steel 316 in concrete pore model solutions. J. Electroanal. Chem..

[40] Veleva L., Alpuche-Aviles M.A., Graves-Brook M.K., Wipf D.O. (2005). Voltammetry and surface analysis of AISI 316 stainless steel in chloride-containing simulated concrete pore environment. J. Electroanal. Chem..

[41] Lambert P., Page C.L., Vassie P.R.W. (1991). Investigations of reinforcement corrosion. 2. Electrochemical monitoring of steel in chloride-contaminated concrete. Mater. Struct..

[42] Mangat P.S., Khatib M., Mollay B.T. (1994). Microstructure, chloride diffusion and reinforcement corrosion in blended cement paste and concrete. Cem. Concr. Compos..

[43] Oh B.H., Jang S.Y., Shin Y.S. (2003). Experimental investigation of the threshold chloride concentration for corrosion initiation in reinforced concrete structures. Mag. Concr. Res..

[44] Glass G.K., Buenfeld N.R. (2000). The influence of chloride binding on the chloride induced corrosion risk in reinforced concrete. Corrosion Sci..

[45] Tomas M.D.A., Hooton R.D., Scott A., Zibara H. (2012). The efect of supplementary cementitious materials on chloride binding in hardened cement paste. Cem. Concr. Res..

[46] Dousti A., Beaudoin J.J., Shekarchi M. (2017). Chloride binding in hydrated MK, SF and natural zeolite-lime mixtures. Constr. Build. Mater..

[47] Shi Z., Geiker M.R., De Weerdt K., Østnor T.A., Lothenbach B., Winnefeld F., Skibsted J. (2017). Role of calcium on chloride binding in hydrated Portland cement–metakaolin–limestone blends. Cem. Concr. Res..

[48] Chaparro W.A., Ruiz J.H., Gomez R.D. (2012). Corrosion of reinforcing bars embedded in alkali-activated slag concrete subjected to chloride attack. Mat. Res..

[49] Rasheeduzzafar, Ehtesham Hussain S., Al-Gahtani A.S. (1991). Pore solution composition and reinforcement corrosion characteristics of microsilica blended cement concrete. Cem. Concr. Res..

[50] Wang W., Chen H., Li H., Zhu Z. (2017). Corrosion behavior of steel bars immersed in simulated pore solution of alkali-activated slag mortar. Constr. Build. Mater..

[51] Wang L., Zhan S., Tang X., Xu Q., Qian K. (2019). Pore solution chemistry of calcium sulfoaluminate cement and its effects on steel passivation. Appl. Sci..

[52] Wang L., Jian Q., Zhan S., Song Y., Ruan S. (2024). Chloride-induced corrosion patters of reinforcements in simulated pore solution of calcium sulfoaluminate cement concrete: An analytical study. J. Build. Eng..

[53] Abd El Haleem S.M., Abd El Wanees S., Bahgat A. (2014). Environmental Factors Affecting the Corrosion Behaviour of Reinforcing Steel. V. Role of Chloride and Sulphate Ions in the Corrosion of Reinforcing Steel in Saturated Ca(OH)_2_ solutions. Corros. Sci..

[54] Liu G., Li J., Zhang Y. (2024). Corrosion of carbon steels subjected to chloride and sulfate in simulated concrete pore solutions with different pH. Constr. Build. Mater..

[55] Bonfil D., Veleva L., Feliu S., Escalante-García J.I. (2023). Corrosion Activity of Stainless Steel SS430 and Carbon Steel B450C in a Sodium Silicate Modified Limestone-Portland Cement Extract. Materials.

[56] Bonfil D., Veleva L., Feliu S., Escalante-García J.I. (2022). Corrosion Activity of Carbon Steel B450C and Stainless Steel SS430 Exposed to Extract Solution of a Supersulfated Cement. Materials.

[57] Lopez-Salas J., Escalante-Garcia J.I. (2024). Hybrid binders based on volcanic pumice: Effect of the chemical composition on strength and microstructures. Cem. Concr. Res..

[58] Bonfil D., Veleva L., Escalante-Garcia J.I. (2024). Effect of a hybrid pumice-portland cement extract on corrosion activity of stainless steel SS304 and carbon mild steel A36. Materials.

[59] Krivenko P.V., Sanytsky M., Kropyvnytska T. (2018). Alkali-sulfate activated blended Portland cements. Solid State Phenom..

[60] (2012). Standard Guide for Laboratory Immersion Corrosion Testing of Metals.

[61] (2017). Standard Practice for Preparing, Cleaning, and Evaluating Corrosion Test Specimens.

[62] Kern M. (1960). The hydration of carbon dioxide. J. Chem. Educ..

[63] Huet B., L’Hostis V., Tricheux L., Idrissi H. (2010). Influence of alkali, silicate and sulfate content of carbonated concrete pore solution on mild steel behavior. Mater. Corros..

[64] Greve-Dierfeld S., Lothenbach B., Vollpracht A., Wu B., Huet B., Andrade C., Medina C., Thiel C., Gruyaert E., Vanoutrive H. (2020). Understanding the carbonation of concrete with supplementary cementitous materials. A critical review by RILEM TC 281-CCC. Mater. Struct..

[65] Peng Y., Liu L., Wang S., Lin Y., Sun Y., Xia R. (2018). Effect of simulated pore solution on passivation characteristic of P110 steel. J. Pet. Sci. Eng..

[66] Zhang Q., Meng X., Li X., Wu L., Suo X., Cao H. (2024). Effect of anions on the anodic dissolution ofi ron: An electrochemical and density functional theory stude. Corros. Sci..

[67] Liu G., Li M., Yang L., Liu C., Zhang Y. (2024). Electrochemical dielectic response of steel corrosion induced by chloride in simulated concrete pore solution. J. Sustain. Cem.-Based Mater..

[68] Ghods P., Isgor O.B., McRae G., Miller T. (2009). The effect of concrete pore solution composition on the quality of passive oxide films on black steel reinforcement. Cem. Concr. Compos..

[69] Krishna Vigneshwaran K.K., Permeh S., Echeverría M., Lau K., Lasa I. (2018). Corrosion of post-tensioned tendons with deficient grout, part 1: Electrochemical behavior of steel in alkaline sulfate solutions. Corrosion.

[70] Li K., Yang L., Wang X., Huang Y. (2019). Influence of SO_4_^2−^ on the corrosion behavior of Q235B steel bar in simulated pore solution. Acta Metall. Sin..

[71] Abd El Haleem S.M., Abd El Wanees S., Abd El Aal E.E., Diab A. (2010). Environmental factor affecting the corrosion behavior of reinforcing steel II. Role of some anions in the initiation and inhibition of pitting corrosion of steel in Ca(OH)_2_ solution. Corros. Sci..

[72] Galvele J.R. (1976). Transport Processes and the mechanism of pitting of metals. J. Electrochem..

[73] Ai Z., Sun W., Jiang J., Song D., Ma H., Zhang J., Wang D. (2016). Passivation Characteristics of Alloy Corrosion-Resistant Steel Cr10Mo1 in Simulating Concrete Pore Solutions: Combination Effects of pH and Chloride. Materials.

[74] Addari D., Elsener B., Rossi A. (2008). Electrochemistry and surface chemistry of stainless steel in alkaline media simulating con-crete pore solutions. Electrochim. Acta.

[75] Luo H., Su H., Dong C., Xiao K., Li X. (2015). Electrochemical and passivation behavior investigation of ferritic stainless steel in simulated concrete pore media. Data in Brief..

[76] Tian Y., Dond C., Wang G., Cheng X., Li X. (2020). The effect of nickel on corrosion behavior of high-strength low alloy steel bar in simulated concrete pore solution. Constr. Build. Mater..

[77] Wahlqvist M., Shchukarev A. (2007). XPS spectra and electronic structure of Group IA sulfates. J. Electron. Spectrosc..

[78] Moulder J.F., Stickle W.F., Sobol P.E., Bomben K.D. (1995). Handbook of X-Ray Photoelectron Spectroscopy: A Reference Book of Standard Spectra for Identification and Interpretation of XPS Data.

[79] Freire L., Catarino M.A., Godinho M.I., Ferreira M.J., Ferreira M.J., Ferreira M.G.S., Simoes A.M.P., Montemor M.F. (2012). Electrochemical and analytical investigation of passive film formed on stainless steel in alkaline media. Cem. Concr. Comp..

[80] Gardin E., Zanna S., Seyeux A. (2018). Comparative study of the surface oxide films on lean duplex and corresponding single phase stainless steels by XPS and ToF-SIMS. Corros. Sci..

[81] Luo H., Su H., Dong C., Li X. (2017). Passivation and electrochemical behavior of 316L stainless steel in chlorinated simulated concrete pore solution. Appl. Surf. Sci..

[82] Abreu C.M., Cristobal M.J., Losada R., Nóvoa X.R., Pena G., Pérez M.C. (2006). The effect of Ni in the electrochemical properties of oxide layers grown on stainless steels. Electrochim. Acta.

[83] Vera R., Villarroel M., Carvajal A.M., Vera E., Ortiz C. (2009). Corrosion Products of Reinforcement in Concrete in Marine and Industrial Environments. Mater. Chem. Phys..

[84] Gotić M., Musić S. (2007). FT-IR and FE SEM Investigation of Iron Oxides Precipitated from FeSO4 Solutions. J. Mol. Struct..

[85] Mataferia I.A. (2021). Characterization of Steel Corrosion Products in Reinforced Concrete. Master’s Thesis.

[86] Antunes R.A., Costa I., Araujo D. (2003). Characterization of Corrosion Products Formed on Steels in the First Months of Atmospheric Exposure. Mater. Res..

[87] Duffó G.S., Worris W., Raspini I., Saragovi C. (2004). A study of steel rebars embedded in concrete during 65 years. Corros. Sci..

[88] Rémazeilles C., Refait P. (2007). On the formation of B-FeOOH (akaganeite) in chloride-containing environments. Corros. Sci..

[89] Freire L., Novoa X.R., Montemor M.F., Carmezin M.J. (2009). Study of passive films formed on mild steel in alkaline media by the application of anodic potentials. Mater. Chem. Phys..

[90] Ghods P., Burkan Isgor O., Bensebaa F., Kingston D. (2012). Angle-resolved XPS of carbon steel passivity and chloride-induced despassivation in simulated concrete pore solution. Corros. Sci..

[91] Burak Gunay H., Ghods P., Burkan Isgor O., Carpenter G.J.C., Wu X. (2013). Characterization of atomic structure oxide films on carbon steel in simulated concrete pore solutions using EELS. Appl. Surf. Sci..

[92] Veleva L. Phase transformation of iron hydroxide in the corrosion products formed in humid tropical climate. Proceedings of the Corrosion 2003.

[93] Tolulope Loto R. (2015). Pitting corrosion evaluation and inhibition of stainless steels: A review. J. Mater. Environ. Sci..

[94] Jiang J.-y., Liu Y., Chu H.-y., Wang D., Ma H., Sun W. (2017). Pitting Corrosion Behaviour of New Corrosion-Resistant Reinforcement Bars in Chloride-Containing Concrete Pore Solution. Materials.

[95] Sun Y., Tan X., Lan R., Ran G., Li J., Jiang Y. (2024). Mechanistics of inclusion-induced pitting of stainless steel: A review. J. Mater. Sci. Technol..

[96] Freire L., Carmezin M.J., Ferreira M.G.S., Montemor M.F. (2011). The electrochemical behaviour of stainless steel AISI 304 in alkaline solution with different pH in the presence of chlorides. Electrochim. Acta.

[97] Jiang J.-Y., Wang D., Chu H.-Y., Ma H., Liu Y., Gao Y., Shi J., Sun W. (2017). The Passive Film Growth Mechanism of New Corrosion-Resistant Steel Rebar in Simulated Concrete Pore Solution: Nanometer Structure and Electrochemical Study. Materials.

[98] Sarango de Souza J., De Oliveira L.A., Sayeg I.J., Antunes R.A. (2017). Electrochemical study of the AISI 409 ferritic stainless steel: Passive film stability and pitting nucleation and growth. Mater. Res..

[99] Orazem M.E., Frateur I., Tribollet B., Vivier V., Marcelin S., Pebere N., Bunge A.L., White E.A., Riemer D.P., Musiani M. (2013). Dielectric properties of materials showing constant-phase-element (CPE) impedance response. J. Electrochem. Soc..

[100] Hirschorn B., Orazem M.E., Tribollet B., Vivier V., Frateur I., Musiani M. (2010). Determination of effective capacitance and film thickness from constant-phase element parameters. Electrochim. Acta.

[101] Ji H., Tian Y., Zhao R., Jin N., Tian Z., Yan D., Ye H. (2020). Passivation and depassivation og HPB335 Carbon steel in simulated concrete pore solution. Int. J. Electrochem. Sci..

[102] Saura P., Zornoza E., Andrade C., Ferrandiz-Mas V., Garcés P. (2022). Composition of Corroded Reinforcing Steel Surface in Solutions Simulating the Electrolytic Environments in the Micropores of Concrete in the Propagation Period. Materials.

[103] Poursaee A., Hansson C.M. (2017). Reinforcing steel passivation in mortar and pore solution. Cem. Concr. Res..

